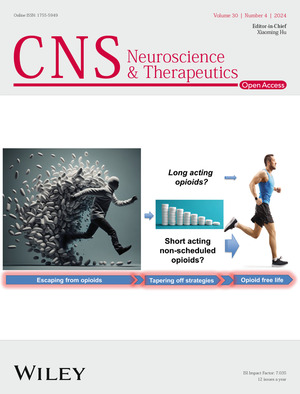# Front Cover

**DOI:** 10.1111/cns.14733

**Published:** 2024-04-16

## Abstract

The cover image is based on the Commentary *Non‐scheduled short acting opioid to taper off opioids?* by Renyu Liu et al., https://doi.org/10.1111/cns.14705.